# Exceptional Complete Functional Recovery After Severe Multitissue Elbow Trauma in a Suicide Attempt Context: A Case Report

**DOI:** 10.7759/cureus.95071

**Published:** 2025-10-21

**Authors:** Mohamed Harmouche, Mohammed Maroc, Abdelilah Rhoul, Younes EL Anbari, Ahmed Amine EL Oumri

**Affiliations:** 1 Physical Medicine and Rehabilitation, Faculty of Medicine and Pharmacy, Mohammed I University, Oujda, MAR; 2 Physical Medicine and Rehabilitation, Mohammed VI University Hospital Center, Oujda, MAR; 3 Physical Medicine and Rehabilitation, Faculty of Medicine and Pharmacy, Sultan Moulay Slimane University, Beni-Mellal, MAR; 4 Physical Medicine and Rehabilitation, Beni-Mellal University Hospital Center, Beni-Mellal, MAR

**Keywords:** brachial artery injury, dash score, elbow trauma, functional outcome, median nerve neurotmesis, nerve recovery, physical medicine and rehabilitation, self-harm, suicide attempt

## Abstract

Complex multitissue upper extremity trauma represents a major therapeutic challenge, particularly in an unfavorable psychiatric context. We report the case of a 33-year-old man admitted for self-inflicted elbow trauma combining complete brachial artery transection with acute ischemia, median nerve neurotmesis, distal biceps tendon rupture, and frontal cerebral contusion. Emergency multidisciplinary surgical management (four-hour delay) included brachial-to-brachial bypass using reversed saphenous vein, end-to-end nerve suture, and tendon repair. Initial assessment revealed complete median nerve palsy (thumb-index distal interphalangeal flexion 0/5, opposition 0/5) with a Disabilities of the Arm, Shoulder and Hand (DASH) score of 78 and poor therapeutic compliance. A structured three-phase rehabilitation program over 12 months, combining physiotherapy, electrotherapy, and occupational therapy, achieved remarkable recovery demonstrated by near-normalized electrophysiological parameters (motor amplitude 3.2 mV, conduction velocity 69.6 m/s) and complete functional recovery with a final DASH score of 18. This exceptional case demonstrates that near-complete recovery is possible after severe nerve trauma, even in a suicide attempt context with unfavorable initial compliance, highlighting the crucial importance of optimal early surgical management and prolonged personalized physical medicine and rehabilitation program.

## Introduction

Complex multitissue injuries of the upper extremity represent a major therapeutic challenge, with less than 50% of patients recovering satisfactory function after nerve repair [1,2]. These injuries affect 2.8% of polytrauma patients and can result in potentially devastating functional consequences [3]. Self-inflicted upper extremity trauma constitutes a rare clinical entity (1.6% of trauma admissions), predominantly involving the wrist [4,5]. Injuries combining brachial artery transection and median nerve neurotmesis at the elbow level are exceptionally reported in the literature.

Complete neurotmesis presents the most severe prognosis according to Seddon's classification [6], with satisfactory functional recovery rates below 50% [2]. Psychological factors associated with suicide attempts further complicate the prognosis [7]. We report an exceptional case of complete functional recovery after severe multitissue elbow trauma in a suicidal context, illustrating the effectiveness of early surgical management followed by a structured rehabilitation program.

## Case presentation

We report the case of a 33-year-old right-handed unemployed male, living in precarious socioeconomic conditions with active chronic alcohol and tobacco use. The patient had no significant surgical history and was not receiving any addiction treatment prior to the trauma.

**Figure 1:**
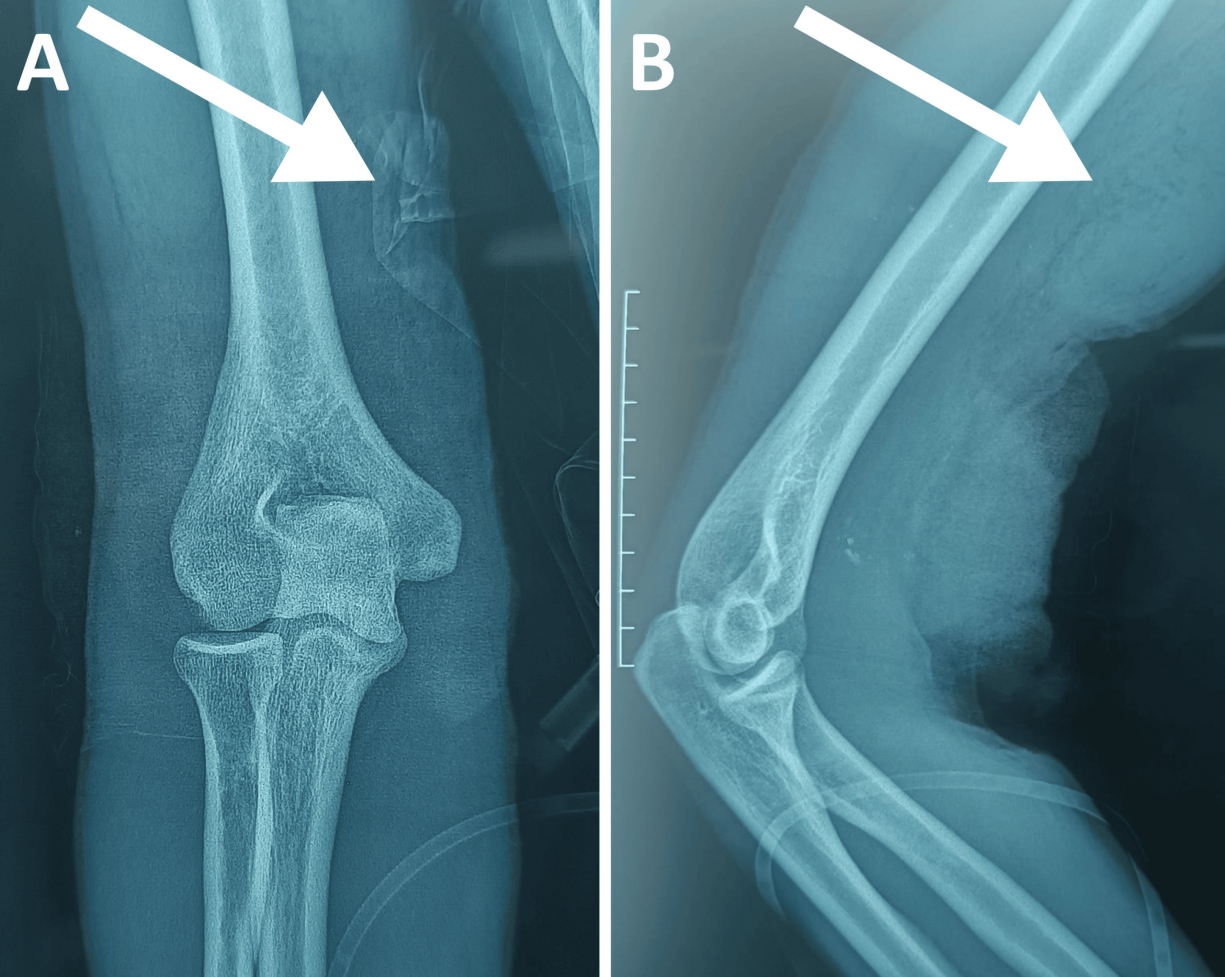
Initial radiological assessment (July 2024) Anteroposterior (A) and lateral (B) radiographs of the right elbow obtained three hours post trauma. (A) Anteroposterior view shows no fracture or dislocation. Note the radiopaque material (white arrow) representing hemostatic dressings and soft tissue loss at the medial wound site. (B) Lateral view confirms intact bony anatomy with proximal soft tissue retraction (white arrow) suggesting distal biceps tendon rupture.

In July 2024, the patient was admitted to the emergency department three hours after sustaining complex multi-tissue right elbow trauma from a broken glass laceration in a suicide attempt. Initial examination revealed an intoxicated patient presenting with severe hemorrhagic shock, with blood pressure of 60/30 mmHg and tachycardia of 140 beats per minute. Vascular examination revealed a deep wound on the medial aspect of the distal third of the right arm with active bleeding and hematoma. The right upper extremity showed acute ischemia characterized by cyanosis and distal coldness, absent radial and ulnar pulses confirmed by absent Doppler flow, and digital saturation collapsed at 80%. Standard elbow radiographs revealed no bone abnormalities (Figure 1).

The complete injury assessment, established after hemodynamic stabilization and surgical exploration, revealed an exceptional combination of major injuries detailed in Table 1. Complete brachial artery transection was responsible for active hemorrhage and distal ischemia. Associated injuries included complete median nerve neurotmesis at the elbow level, complete distal biceps tendon rupture, and extensive cutaneous wound exposing vital/neurovascular structures. Preoperative brain computed tomography scan, motivated by associated head trauma, revealed frontal contusion requiring prophylactic antiepileptic treatment with carbamazepine for one month.

**Table 1:** Comprehensive initial multitissue injury assessment (July 2024) Documentation of all injured structures identified during emergency evaluation and surgical exploration. Lists anatomical structures, specific injuries, and their immediate functional consequences requiring multidisciplinary surgical management within four hours.

Anatomical structure	Identified injury	Functional consequences
Vascular	Complete brachial artery transection	Acute ischemia, active hemorrhage
Neurological	Complete median nerve neurotmesis	Sensory-motor deficit in median territory
Musculotendinous	Complete distal biceps tendon rupture	Elbow flexion deficit
Cutaneous	Extensive wound medial elbow	Exposure of noble structures
Cerebral	Frontal contusion (computed tomography)	Prophylactic carbamazepine for one month

Emergency surgical management was performed within a remarkably short four-hour window, requiring close collaboration between vascular and orthopedic surgery teams. Vascular reconstruction consisted of brachial-to-brachial bypass using an 8-cm reversed saphenous vein autograft. After hemorrhage control with an Esmarch bandage and systemic heparinization, proximal and distal end-to-end anastomoses were performed using 6/0 Prolene suture. Graft patency was verified by Fogarty catheter passage, retrieving no thrombus, with immediate recovery of distal pulses. Nerve repair was performed by direct end-to-end median nerve suture; the absence of substance loss eliminated the need for nerve grafting. Epiperineurial suture was performed with 6/0 Prolene monofilament. Distal biceps tendon repair was achieved by end-to-end suture with cardinal solidarization stitches. The procedure concluded with careful debridement and layered skin closure. The immediate postoperative course was uncomplicated with maintained satisfactory distal vascularization.

**Figure 2:**
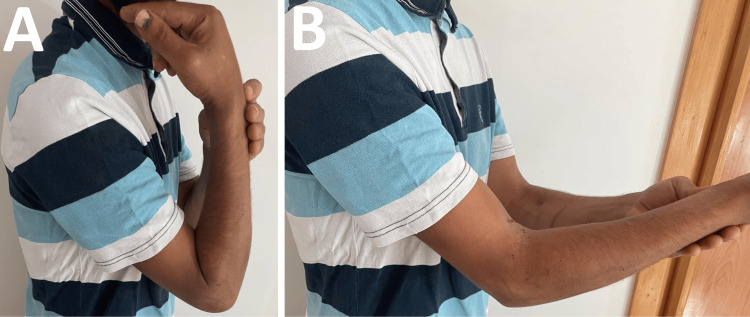
Clinical photographs at one month postoperative (August 2024) (A) Maximum elbow flexion limited to 130° (normal 150°). (B) Extension deficit of -60°, showing significant flexion contracture typical after complex elbow trauma. These limitations corresponded to DASH score of 78/100. DASH: Disabilities of the Arm, Shoulder and Hand

Initial assessment at one month postoperatively shows limitation of elbow flexion to 130° (Figure 2A) and extension to -60° (Figure 2B) in pronation and supination. Clinical examination revealed complete paralysis of thumb and index distal interphalangeal flexion, rated 0/5 on the Medical Research Council (MRC) scale, as well as total thumb opposition paralysis indicating complete thenar eminence involvement. These deficits rendered functional thumb-index pinch impossible. Sensory examination showed permanent paresthesias affecting the entire median nerve territory, involving the first three finger pulps and radial half of the fourth finger.

Initial functional assessment using validated outcome measures revealed severe disability with a Disabilities of the Arm, Shoulder and Hand (DASH) score of 78, indicating significant functional impairment in activities of daily living (ADL) [8]. During this initial period, we encountered poor therapeutic compliance; the patient showed minimal engagement in the rehabilitation program and categorically refused psychiatric or addiction follow-up despite repeated recommendations.

**Figure 3:**
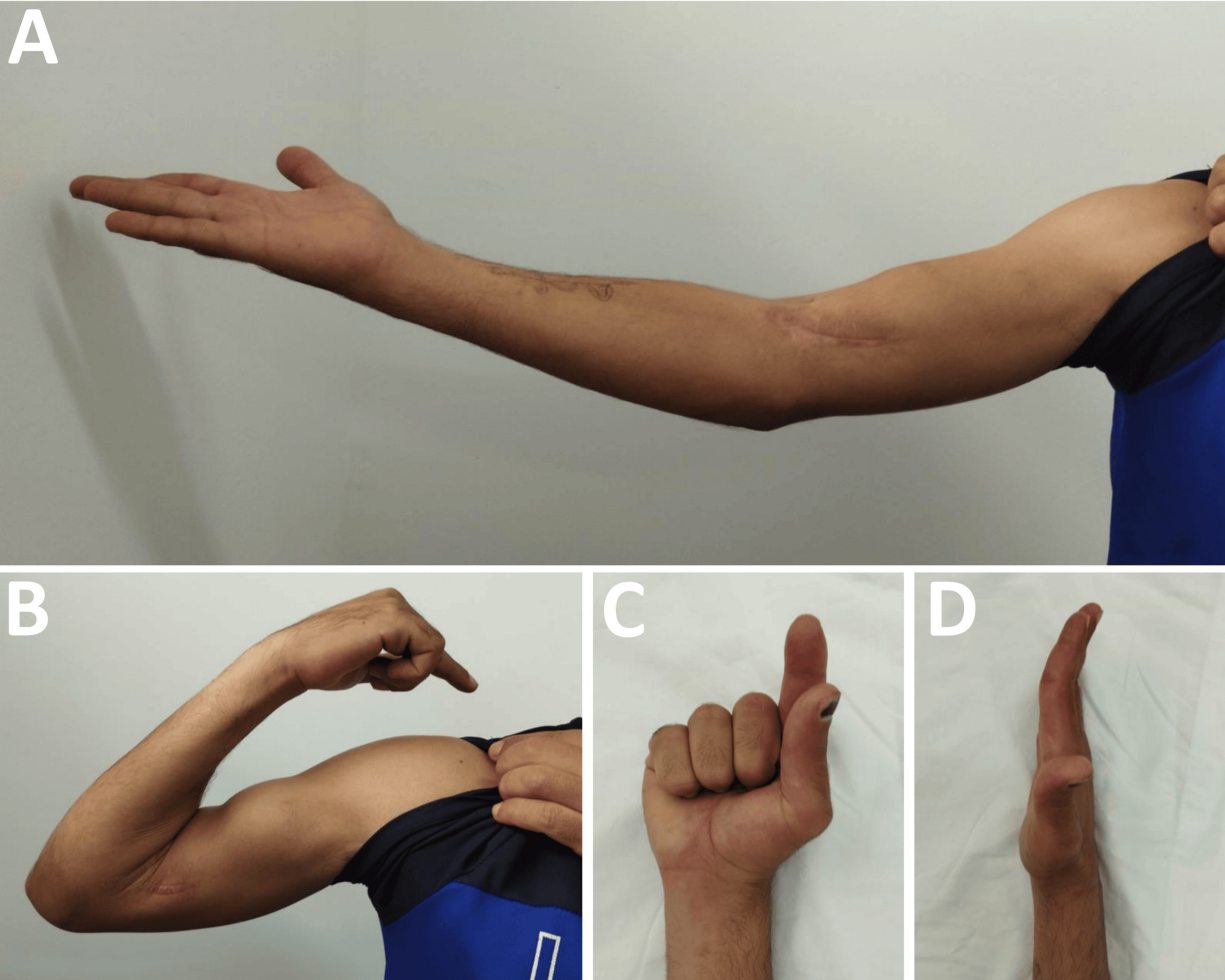
Clinical photographs at three months postoperatively (October 2024) (A) Elbow extension improved to -30° (from initial -60°). (B) Preserved elbow flexion at 150° with healed surgical scar. (C) Complete paralysis of thumb and index finger flexion (0/5 on Medical Research Council scale) due to median nerve injury. (D) Compensatory metacarpophalangeal hyperextension during finger extension, typical of median nerve palsy.

The three-month postoperative assessment revealed major neurological sequelae summarized in Table 2. Analytical examination revealed complete paralysis of the thumb and index distal interphalangeal flexion, rated 0/5 on the MRC scale, as well as total thumb opposition paralysis, indicating complete thenar eminence involvement. These deficits rendered functional thumb-index pinch impossible. Sensory examination showed permanent paresthesias affecting the entire median nerve territory, involving the first three finger pulps and radial half of the fourth finger. Joint examination revealed significant elbow stiffness with preserved flexion at 150° (Figure 3A) but extension limited to -30° (Figure 3B), associated with slight pronation limitation. Inability to flex the distal interphalangeal joints of the thumb and index finger was noted (Figure 3C), with a tendency toward metacarpophalangeal hyperextension during active finger extension (Figure 3D). Neurological examination found a positive Tinel sign at the suture site at the elbow, an encouraging sign indicating early axonal regeneration. Distal vascularization remained satisfactory with well-perceived radial and ulnar pulses.

**Table 2:** Detailed clinical findings at three-month assessment (October 2024) Systematic evaluation documenting persistent neurological and functional deficits three months post-surgery. Motor strength assessed using MRC scale (0-5), sensory deficits mapped by territory, joint range measured by goniometry, and functional status quantified by DASH score (78/100 indicating severe disability). DIP: distal interphalangeal ; D1, D2, D3, D4: digit 1 (thumb), digit 2 (index), digit 3 (middle finger), digit 4 (ring finger); MCP: metacarpophalangeal; DASH: Disabilities of the Arm, Shoulder and Hand; ADL: activities of daily living; MRC: Medical Research Council

Domain	Observed deficits	Clinical signs
Motor	DIP thumb paralysis 0/5, DIP index paralysis 0/5, Thumb opposition paralysis 0/5, according to the MRC scale.	Impossible thumb-index pinch, Flat hand
Sensory	D1-D2-D3 pulp anesthesia, D4 hemi-radial hypoesthesia	Permanent paresthesias, Positive Tinel at anterior elbow
Joint	Elbow flexion 150 degrees, Extension -30 degrees, Limited pronation	Elbow and finger stiffness, MCP hyperextension
Functional	DASH score: 65	ADL dependence, Refusal of psychiatric/addiction follow-up

**Figure 4:**
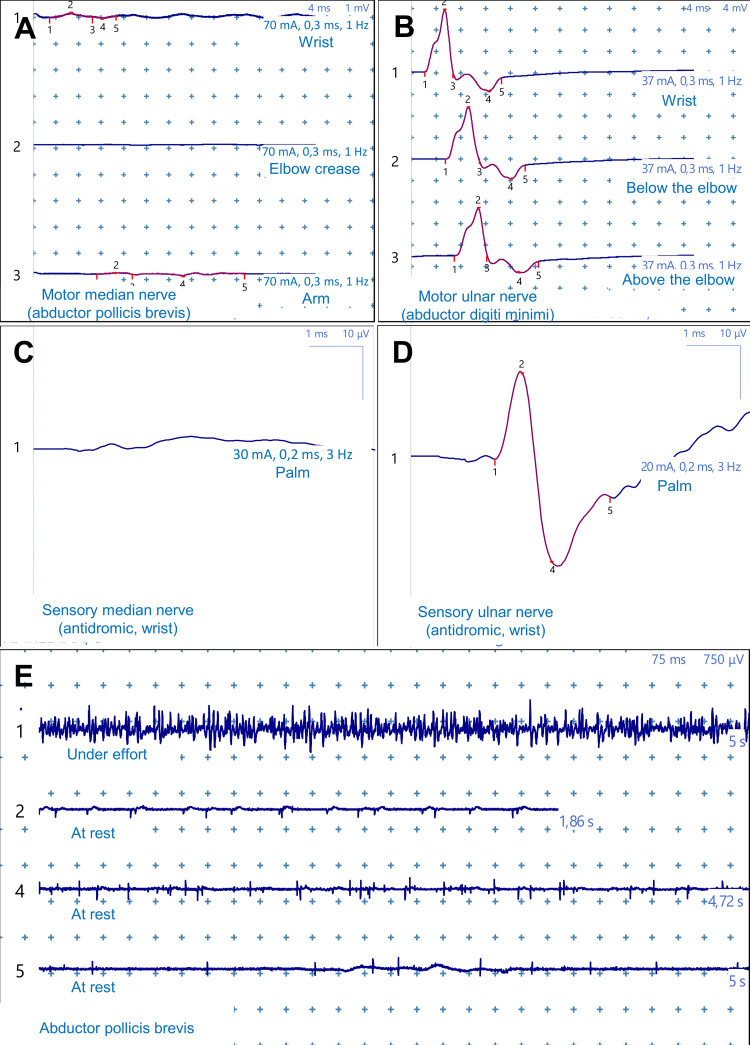
Electroneuromyography at three months showing complete median neurotmesis (October 2024) (A) Absent median motor response at wrist, elbow and arm. (B) Normal ulnar motor response (12 mV) excluding generalized neuropathy. (C) Absent median sensory response. (D) Normal ulnar sensory response confirming isolated median pathology. (E) Needle electroneuromyography showing fibrillation potentials and no voluntary units in abductor pollicis brevis, indicating active denervation.

Electroneuromyography (ENMG) performed in October 2024 (Figure 4), three months post trauma, confirmed nerve injury severity. Nerve conduction studies showed a complete absence of motor and sensory response of the right median nerve (Figures 4A, 4C), contrasting with strictly normal electrophysiological parameters for the ipsilateral ulnar nerve (Figures 4B, 4D). Needle ENMG revealed intense pathological spontaneous activity in flexor carpi radialis and abductor pollicis brevis, with a poor microvolt neurogenic pattern for the latter and a rich neurogenic pattern with high amplitude potentials for flexor carpi radialis (Figure 4E). These findings confirmed complete right median nerve neurotmesis at elbow level with active denervation of tributary muscles.

Clinical evaluation at three months showed modest improvement in elbow range of motion with a persistent -30° extension deficit. Fine motor deficits remained unchanged. However, the Tinel sign had progressed to the upper third of the forearm, indicating nerve regeneration progression at approximately 1 mm per day. The functional score showed modest improvement with DASH decreasing to 65.

A significant turning point in evolution was observed at the December 2024 assessment. Clinical examination revealed partial thumb opposition recovery, allowing functional thumb-index pinch initiation. The Tinel sign had continued progression, reaching the middle third of the forearm. Electrophysiological evolution was particularly encouraging with reappearance of motor potentials still of low amplitude (0.31 mV at wrist and 0.15 mV at arm) but with measurable conduction velocity at 45.1 m/s. Sensory conduction also showed recovery signs with reappearance of sensory potentials of 0.41 µV amplitude and conduction velocity of 34.0 m/s. This neurophysiological improvement was accompanied by significant functional progress, with DASH score improving to 45. Complete evolution of electrophysiological parameters throughout follow-up is summarized in Table 3, showing remarkable nerve recovery progression. Concurrently, detailed clinical-functional evolution in Table 4 illustrates a correlation between electrophysiological data and functional recovery.

**Table 3:** Longitudinal electrophysiological parameters documenting progressive nerve regeneration Serial electroneuromyography at three, six, and 12 months showing evolution from complete absence of responses to near-normal values (motor amplitude 3.2 mV, conduction velocity 69.6 m/s). Progressive Tinel sign advancement correlated with 1 mm/day regeneration rate. MCV: motor conduction velocity; SCV: sensory conduction velocity; ENMG: electroneuromyography; SA++: spontaneous activity; FCR: flexor carpi radialis; APB: abductor pollicis brevis; N: normal; NN: near-normal

Date	Motor conduction	Sensory conduction	Needle ENMG	Interpretation
At three months post-operative	Total absence of response	Total absence of response	SA++ FCR and APB, Neurogenic pattern	Complete neurotmesis, Active denervation
At six months post-operative	Amplitude: 0.31 mV (wrist), MCV: 45.1 m/s	Amplitude: 0.41 µV, SCV: 34.0 m/s	Not performed	Beginning reinnervation, Tinel mid-forearm
At twelve months post-operative	Amplitude: 3.2 mV (NN), MCV: 69.6 m/s (N)	Amplitude: 1.1 µV (NN), SCV: 54.8 m/s (N)	Not performed	Near-complete recovery

**Table 4:** Correlation between clinical and functional recovery over 12 months Parallel evolution of joint mobility, motor recovery, Tinel sign progression, and DASH scores over 12 months. Shows progression from complete median paralysis and severe disability (DASH 78/100) to full recovery (DASH 18/100). F: flexion; E: extension; DASH: Disabilities of the Arm, Shoulder and Hand

Time period	Joint range of motion	Motor recovery	Tinel progression	DASH score
At one month post-operative	F: 130° / E: -60°	Complete median paralysis	Elbow	78
At three months post-operative	F: 150° / E: -30°	Persistent paralysis	Upper 1/3 forearm	65
At six months post-operative	F: 150° / E: -20°	Partial thumb opposition	Mid 1/3 forearm	45
At twelve months post-operative	F: 150° / E: 0°	Complete recovery	Wrist	18

**Figure 5:**
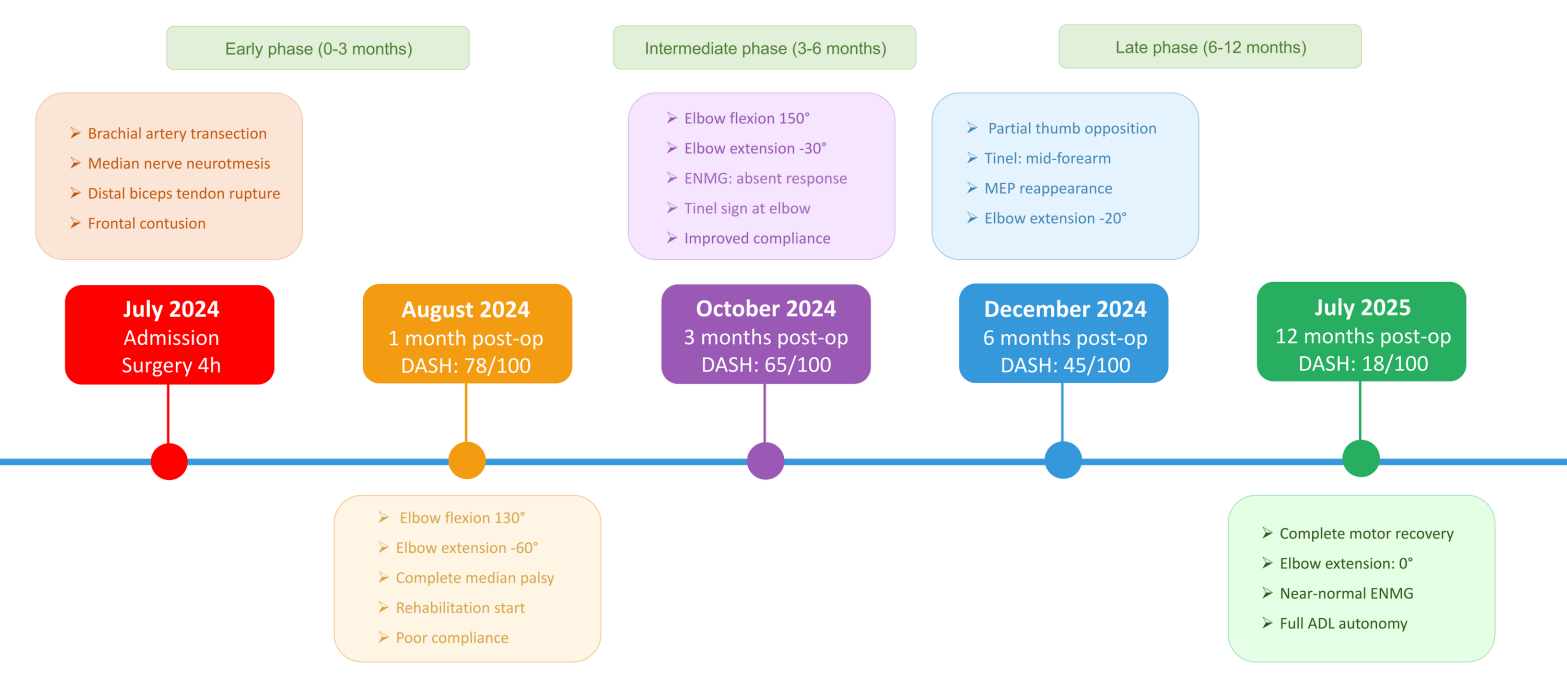
Clinical Timeline of Multitissue Elbow Trauma Recovery Following Suicide Attempt Comprehensive timeline illustrating the 12-month recovery course of a 33-year-old male following multitissue elbow trauma. The timeline presents five evaluation points with clinical and functional outcomes. Initial emergency phase (July 2024) shows severe injuries managed surgically within four hours. Progressive recovery is demonstrated through: DASH scores improving from 78/100 (severe disability) to 18/100 (near-normal function); elbow range of motion normalizing from significant contracture (flexion 130°/extension -60°) to full extension; motor recovery progressing from complete median nerve palsy through partial thumb opposition to complete recovery; Tinel sign advancement tracking nerve regeneration from elbow to wrist. Three rehabilitation phases guide treatment progression. Electrophysiological studies at three months showed absent responses, with MEP reappearance at six months confirming reinnervation. DASH: Disabilities of the Arm, Shoulder and Hand; ENMG: electroneuromyography; MEP: motor evoked potentials; ADL: activities of daily living

The patient's clinical course from initial trauma to complete recovery is illustrated in Figure 5, providing a comprehensive visual timeline of functional outcomes and key milestones over the 12-month follow-up period. This timeline demonstrates the progressive improvement in DASH scores from 78/100 at one month to 18/100 at 12 months, correlating with the structured three-phase rehabilitation protocol described in Table 5.

It is important to emphasize that the patient's therapeutic compliance markedly improved after the third month, coinciding with the appearance of the first objective signs of neurological recovery. This improvement in rehabilitation program adherence likely played a determining role in subsequent favorable evolution.

**Figure 6:**
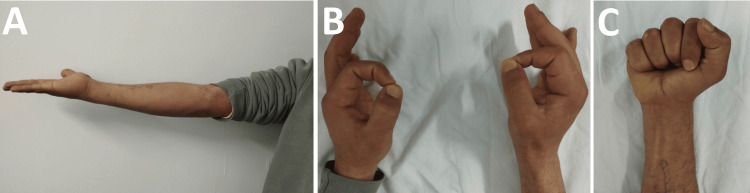
Clinical photographs at 12 months showing complete functional recovery (July 2025) (A) Full elbow extension (0°) achieved, resolving initial -60° contracture. (B) Recovered thumb-index opposition demonstrating successful median nerve regeneration. (C) Normal finger flexion including thumb and index distal interphalangeal joints. Final DASH score 18/100. DASH: Disabilities of the Arm, Shoulder and Hand

Final assessment performed in July 2025, one year after initial trauma, revealed remarkable functional recovery. Clinical examination found only mild residual paresthesias in the median nerve territory, with the patient having recovered complete motor function. Finger flexion demonstrated good recovery of thumb and index distal interphalangeal flexion (Figure 6C), with total recovery of thumb opposition allowing functional thumb-index pinch, symmetrical to the contralateral side (Figure 6B). Elbow range of motion was normalized with complete extension at 0° (Figure 6A) and preserved flexion at 150°. Grip function was symmetrical to the contralateral side, allowing complete autonomy for all ADL. Final clinical photographs demonstrate this exceptional recovery (Figure 6).

**Figure 7:**
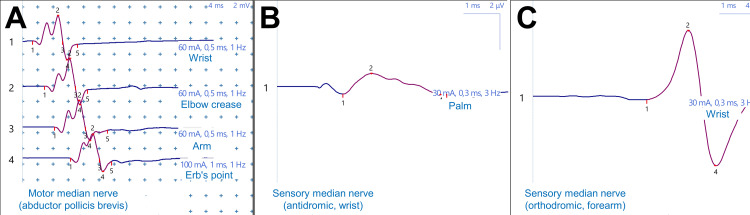
Electroneuromyography at 12 months showing near-complete recovery (July 2025) (A) Near-normalized median motor response (3.2 mV amplitude) without conduction block. (B) Recovered median sensory response (1.1 μV, 54.8 m/s). (C) Normal sensory conduction at forearm level. Near-complete electrophysiological recovery achieved.

Final electrophysiological evolution was exceptional with near-complete normalization of all median nerve parameters (Figure 7). Motor amplitude at the wrist reached 3.2 mV, approaching normal limits, with near-normalized conduction velocities both distally (69.6 m/s) and proximally at Erb's point (71.3 m/s). Amplitude was also preserved throughout the nerve course, measured at 3.5 mV at the elbow crease and 3.4 mV at the arm, demonstrating homogeneous recovery without residual conduction block. Sensory conduction was nearly normalized with amplitude at 1.1 µV and conduction velocity at 54.8 m/s. This remarkable neurophysiological recovery translated into excellent final functional outcomes with a DASH score of 18, indicating near-normal upper extremity function and supporting potential return to work.

Rehabilitation management was structured around a protocol organized in three distinct phases, progressively adapted to patient clinical evolution and therapeutic compliance improvement. The early phase primarily targeted post-traumatic edema control, joint range of motion maintenance, and perineural adhesion prevention, crucial elements for creating favorable tissue conditions for nerve regeneration. The intermediate phase, beginning with appearance of first reinnervation signs, progressively introduced adapted muscle strengthening and specific sensory reeducation according to neuronal plasticity principles. The late phase focused on functional optimization with intensive work on strengthening, coordination, and readaptation to specific professional gestures. A personalized daily self-program systematically complemented supervised sessions at each phase, maximizing neuromotor stimulation and patient active involvement in recovery. Detailed modalities of this protocol are presented in Table 5.

**Table 5:** Evidence-based three-phase rehabilitation protocol Structured rehabilitation program adapted to nerve regeneration stages: early phase (one to three months) for tissue protection, intermediate phase (three to six months) for reinnervation, and late phase (6-12 months) for functional optimization. Each phase includes specific techniques, frequency, and home exercises. TENS: transcutaneous electrical nerve stimulation; ROM: range of motion

Phase	Period	Frequency	Therapeutic objectives	Techniques used	Daily self-program
Early	Months 1 to 3	3×/week 45 min	Edema control, Maintain joint ROM, Prevent adhesions, Analgesia, Maintain motor pattern	Gentle passive then active-assisted mobilization, Manual centripetal lymphatic drainage, Analgesic TENS 80 Hertz 20 min on median nerve pathway, Cryotherapy 15 min 3x/day, Gentle stretching postures, Biceps/triceps isometric contractions sets of 10x5 seconds, Scar massage	Active self-mobilization 5x/day, Night resting splint wear, Limb elevation, Pendulum exercises, Isometric contractions 3x/day
Intermediate	Months 3 to 6	3×/week 45 min	Muscle strength recovery, Sensory reeducation, Joint range of motion gain, Coordination initiation	Progressive strengthening isometric to concentric to eccentric, Muscle electrostimulation 35 Hertz 6 seconds/12 seconds, Dellon method for progressive tactile discrimination, Contrast baths hot/cold, Occupational therapy gross grasp to fine grips, Specific median nerve mobilization, Digital dissociation exercises	Strengthening program with elastic bands, Sensory discrimination exercises 2x/day, Varied texture object manipulation, Multiple daily active stretches
Late	Months 6 to 12	2×/week 60 min	Maximum functional strengthening, Professional readaptation, Muscle endurance, Complete autonomy	Progressive resistance strengthening weights pulleys, Adapted plyometric exercises, Professional gesture simulation tools loads, Proprioceptive work eyes closed unstable surfaces, Complex bimanual coordination, Endurance training long sets, Global neuromotor reprogramming	Adapted strengthening program, Job-specific functional exercises, Complex manual activities, Adapted sport swimming recommended

## Discussion

Our observation presents several remarkable features that merit detailed analysis. The exceptionally favorable recovery observed contrasts with several initially unfavorable prognostic factors, including the psychiatric context of the suicide attempt, the severity of multitissue injuries, and initially poor therapeutic compliance. According to literature, the main determinants of prognosis after peripheral nerve injury include patient age, type of injured nerve, location and extent of injury, repair delay, and surgical technique used [9]. In our case, several elements were particularly favorable: relatively young patient age (33 years), allowing optimal regeneration potential, possibility of performing direct nerve repair without the need for graft due to the absence of substance loss, and especially remarkably short four-hour delay between trauma and surgical management. To our knowledge, no similar cases combining brachial artery transection, median nerve neurotmesis, and distal biceps tendon rupture at the elbow level in the context of a suicide attempt have been previously reported in the literature. This makes direct comparison impossible and highlights the exceptional nature of this complete recovery.

Repair delay constitutes a major independent predictive factor for functional recovery [10]. In this case, ultra-early management minimized nerve retraction and cellular degeneration phenomena, thus optimizing conditions for subsequent axonal regeneration. Exemplary coordination between vascular and orthopedic surgery teams was determined, allowing a global approach to multitissue trauma. Immediate revascularization by saphenous vein bypass not only saved the limb from acute ischemia but also maintained optimal tissue conditions for nerve regeneration, vascularization being an indispensable prerequisite for the nerve repair process [11]. Concomitant repair of all three injured structures (artery, nerve, tendon) in a single operative time presents several advantages: reduction in the number of interventions, decreased infection risk, and especially the possibility of early rehabilitation initiation. This global approach corresponds to current recommendations for complex upper extremity trauma management [12].

The nature of nerve injury also constitutes a determining element. Complete neurotmesis, characterized by total nerve transection including the endoneurium, generally carries the most severe prognosis according to Seddon's classification [13]. However, in our case, the absence of nerve substance loss allowed direct end-to-end suture, a technique associated with the best functional results compared to nerve grafts [14].

The documented electrophysiological evolution in our observation didactically illustrates different phases of peripheral nerve regeneration. Initial total absence of response at three months corresponds to the expected phase of complete Wallerian degeneration downstream from injury [15]. Progressive reappearance of action potentials from the sixth month, with initially very low amplitudes, then progressively normalizing, demonstrates an ongoing reinnervation process [15]. Tinel sign progression from elbow to forearm, then wrist constituted a precious clinical marker, allowing not only anatomical tracking of axonal regrowth progression at the classic rate of 1 mm per day, but also providing the patient with tangible and encouraging proof of ongoing recovery.

A particularly interesting aspect of our observation concerns the evolution of patient therapeutic compliance. Spectacular improvement in rehabilitation program adherence after the third month, precisely coinciding with the appearance of the first objective neurological recovery signs, emphasizes the crucial importance of psychological factors in the recovery process [7]. This observation suggests that patient motivation can be significantly influenced by observation of tangible progress, transforming an initial vicious cycle into a virtuous one. The particular context of a suicide attempt could have constituted an extremely unfavorable prognostic factor, potentially compromising therapeutic adherence and thus chances of recovery. However, the healthcare team's perseverance and continuous adaptation of therapeutic strategies overcame this major obstacle.

The rehabilitation protocol, structured in three phases, played a determining role in functional recovery optimization. Each phase was carefully adapted to the patient's clinical and neurophysiological evolution. The initial phase, focused on complication prevention, maintained favorable tissue conditions despite denervation. The intermediate phase, coinciding with first reinnervation signs, optimized nascent functional recovery through specific occupational therapy work. The late phase, oriented toward professional readaptation, illustrates the importance of long-term vision exceeding simple anatomical recovery to aim for complete socioprofessional reintegration.

Systematic use of the DASH score as a longitudinal assessment tool proved particularly relevant. The 60-point improvement observed far exceeds the minimal clinically significant change established at 10 points in the literature [8,16]. This spectacular improvement demonstrates not only anatomical recovery but especially functional restoration, allowing return to normal life. The socioeconomic impact of this recovery is considerable when considering that forearm nerve injuries result in job loss in nearly a quarter of cases, according to literature data [17].

Despite these exceptional results, several limitations must be emphasized. The isolated nature of this observation does not allow generalization of our conclusions to all similar trauma. Absence of structured psychiatric follow-up, despite our repeated recommendations, represents a missed opportunity for global management optimization. It is possible that adapted psychological support would have allowed earlier improvement in therapeutic compliance and perhaps even further optimization of functional recovery. While a comprehensive cost-benefit analysis was not performed in this case, the return to functional independence (DASH 18/100) suggests that intensive multidisciplinary management may prevent long-term disability costs and improve quality of life, even in challenging psychosocial contexts. These encouraging results nevertheless raise interesting perspectives for the development of standardized management protocols integrating from the outset somatic and psychosocial dimensions of these complex traumas.

This case provides important clinical implications for managing severe upper extremity trauma in suicide attempt contexts. Key success factors include: emergency surgical management within four to six hours to optimize nerve regeneration potential, multidisciplinary coordination between vascular and orthopedic teams for simultaneous repair of all injured structures, early initiation of structured rehabilitation despite initial poor compliance, and persistence in therapeutic approach even when early psychological engagement is limited. These elements should guide protocols for similar complex injuries.

## Conclusions

This observation demonstrates that complete functional recovery is possible after severe multitissue elbow trauma, even in a suicide attempt context with unfavorable initial compliance. The combination of ultra-early multidisciplinary surgery (four hours) and structured rehabilitation program over 12 months allowed exceptional recovery (DASH score, 78-18). Key success factors included emergency surgical management within four to six hours to optimize nerve regeneration potential, simultaneous repair of all injured structures in a single operative session, and persistence with structured rehabilitation despite initially poor therapeutic compliance. The transformation from non-compliance to active participation following visible neurological recovery at three months highlights the importance of maintaining therapeutic engagement during the critical early period.

The exceptional functional recovery emphasizes the importance of persevering in management despite initial obstacles and offers a message of hope for similar trauma. While acknowledging the limitations of a single case report, this observation suggests that aggressive early intervention combined with sustained rehabilitation efforts can achieve outcomes exceeding conventional expectations, even in the challenging context of intentional self-harm. Future prospective studies should investigate optimal management protocols integrating both somatic and psychological interventions to further improve outcomes in this complex patient population.
